# Supplementary material for: Altered Storage and Function of von Willebrand Factor in Human Cardiac Microvascular Endothelial Cells Isolated from Recipient Transplant Hearts
Source: Int J Mol Sci. 2023 Feb 25;24(5):4553. doi: 10.3390/ijms24054553 (PMC10003102; doi:10.3390/ijms24054553)
Supplement: Supplementary file 1 [file ijms-24-04553-s001.zip › ijms-2213287-supplementary.pdf]

# Supplemental data

## Table S1 HMVEC-C (Lonza, CC-7030) inventory

| Part Number | Cell Type | TAN | Lot No. | Age | Sex | Race | Seed Eff. | Viab Excl | Dblg Time | Alco Use | Smok Use | Cell Pass. | Cell Count | QC Eval Medium | Pop. Tot. |
|---|---|---|---|---|---|---|---|---|---|---|---|---|---|---|---|
| CC-7030 | HMVEC-C | 27183 | 0000399195 | 49 Y | M | C | 52 | 86 | 26 | Y | Y | 3 | 737,500 | EGM-2 | 10 |
| CC-7030 | HMVEC-C | 28758 | 0000473429 | 47 Y | M | C | 56 | 91 | 21.8 | Y | Y | 3 | 841,500 | EGM-2 | 10 |
| CC-7030 | HMVEC-C | 28760 | 0000473674 | 35 Y | M | H | 46 | 83 | 21.7 | Y | Y | 3 | 945,500 | EGM-2 | 10 |

## Table S2 Antibodies and dilutions

| Antigen | **Species** | **Manufacturer** | **Clone / catalogue number** | **Dilutions used for ICC** |
|---|---|---|---|---|
| **1 VWF** | Rabbit pAb | DAKO/Agilent Santa Clara, CA USA | A0082 | 1:10000 |
| **2 VWF** | Sheep pAb | Serotec Raleigh, NC, USA | AHP062 | 1:10000 |
| **3 VWFpp** | Rabbit pAb | Harlan UK Ltd | See (Hewlett et al., 2011) | 1:5000 |
| **4 P-selectin** | Mouse pAb | Bio-Rad (Formerly AbD Serotec) | AK6/MCA796 | 1:50 |
| **5 Rab27A** | **rabbit pAb** | Harlan UK Ltd | See (Bierings et al., 2012) | **1:100** |
| **6 Rab27A** | Mouse mAb | BD Transduction Laboratories | R52320 | 1:100 |
| **7 MyRIP** | **Goat pAb** | Abcam | ab10149 | **1:200** |
| **8 Slp4-a** | Rabbit pAb | Atlas Antibodies | HPA001475 | 1:5000 |
| **9 Rab3B** | Mouse mAb | Abnova | H00005865-M01 | 1:1000 |
| **10. tPA** | Sheep mAb | Serotec (Kidlington, UK | Cat No: 9020-0809 | 1:125 |

11. Fluorophore coupled secondary antibodies used in this study were purchased from Jackson ImmunoResearch Europe (Newmarket, UK). The catalogue numbers are: Alexa 488 AffiniPure Donkey Anti-Mouse IgG (715-545-150), Rhodamine Red™-X (RRX) AffiniPure Donkey Anti-Mouse IgG (115-295-003), Alexa 488 AffiniPure Donkey Anti-Rabbit IgG (711-545-152), Rhodamine Red™-X (RRX) AffiniPure Donkey Anti-Rabbit IgG (711-295-152), Alexa 488 AffiniPure Donkey Anti-Sheep IgG (713-545-147). Rhodamine Red™-X (RRX) AffiniPure Donkey Anti-goat IgG.

Antibody Validation

1. Anti-Human VWF (A0082) has been validated for detection of von Willebrand factor in endothelial cells, megakaryocytes and platelets when tested on formalin-fixed, paraffin-embedded normal human tissues (bone marrow, kidney, liver, lung, lymph node, skin and spleen) as described on the Agilent website https://www.agilent.com/en/product/dako-omnis-solution-for-ihc-ish/primary-antibodies-for-dako-omnis/primary-antibodies-(flex-ready-to-use)/von-willebrand-factor-(dako-omnis)-76216 ). We use the Ab extensively in our lab for ICC and WB and it recognises epitope tagged VWF both in fixed and live HUVEC.
2. Anti-human VWF Ab has been verified for detection of VWF by Serotec and in multiple publications as described on the BioRad website https://www.citeab.com/antibodies/111902-ahp062-sheep-anti-human-von-willebrand-factor). We have used the Ab extensively in our lab for ICC and WPB and it recognises epitope tagged VWF in fixed aHUVEC.
3. VWF propeptide antibody was characterised and validated by western blot and ICC in (Hewlett et al., 2011).
4. Anti-CD62P/P-selectin (AK6), Immunofluorescence; has been verified for detection of P-selectin by Serotec labs and in multiple publications as described on the BioRad website (https://www.bio-rad-antibodies.com/monoclonal/human-cd62p-antibody-ak-6-mca796.html?f=purified). In our hands AK6 recognises epitope tagged P-selectin expressed in HUVEC.
5. Characterisation and validation of Rab27A polyclonal antibody is described in (Bierings et al., 2012). The antibody recognises epitope tagged rab27A by western blot and by ICC in situ in PFA fixed endothelial cells. .
6. Mouse anti-Rab27a antibody was validated in (Bierings et al., 2012), where it detected epitope tagged Rab27a iby ICC.
7. Anti-MyRIP antibody was validated by western blot by the manufactures https://www.abcam.com/myrip-antibody-ab10149.html and has been used in several publications successfully to detected the prtein by ICC, as indicated on the manufactires website.
8. Anti-SLP4a detects epitope tagged SLP4a by western blot (https://www.atlasantibodies.com/products/antibodies/primary-antibodies/triple-a-polyclonals/sytl4-antibody-hpa001475/) and by ICC (Bierings et al., 2012).
9. Anti-Rab3B selectively detects epitope tagged rab3B by western blot and ICC (Bierings et al., 2012).
10. Sheep Anti-tPA antibody was validated against eptiptope tagged tPA in (Knipe et al., 2010)

11. Fluorophore coupled secondary antibodies used in this study have been extensively used in our laboratories, validated by the same methods as primary Abs as well as negative control lacking primary antibodies. They are well established and have been widely used.

Software and code

1. OriginLabs Origin 2018 sr1.
2. GraphPad prism 9.0.2 Feb 2021
3. Photoshop CC vr 23.2.2 (2022)
4. MS Office Professional Plus 2019

Figure S1

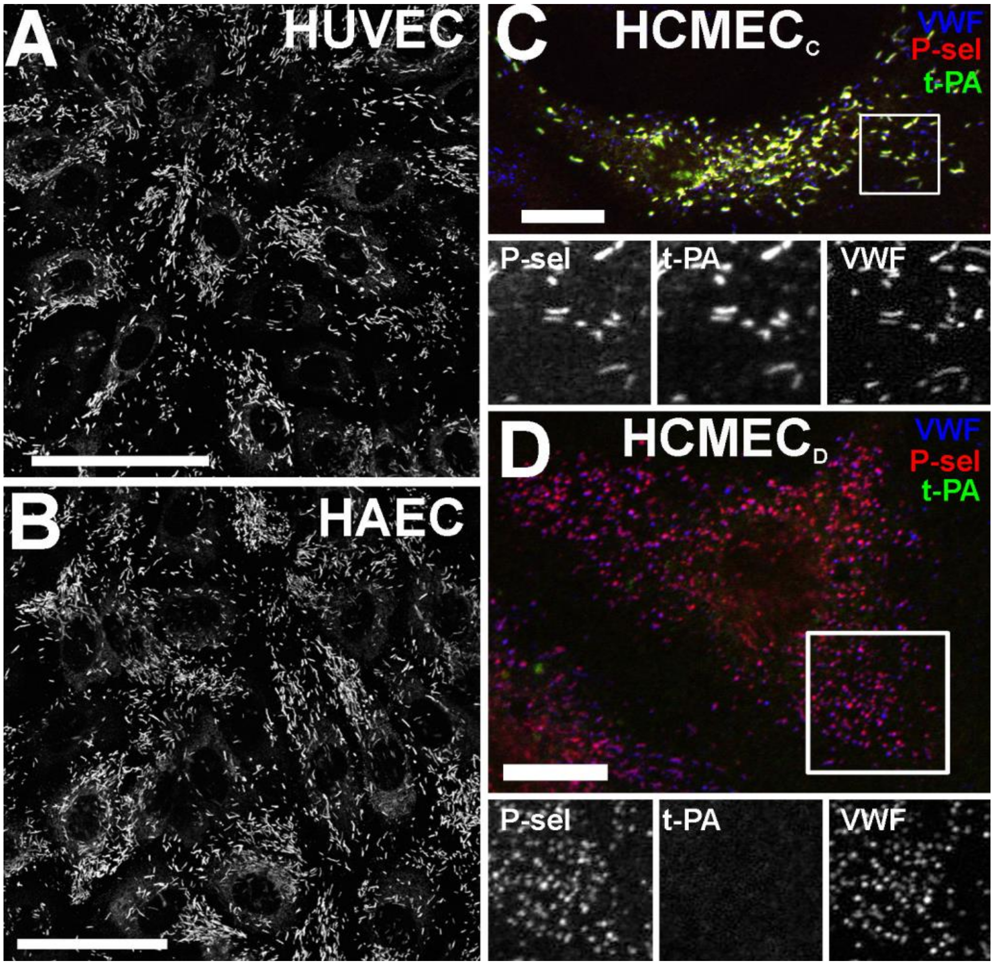


**Figure S1**. **HCMEC$_D$ contain P-selectin but not tissue plasminogen activator (tPA).** Fixed HUVEC (A) and HAEC (B) immunolabeled for endogenous VWF. Scale bars 50μm. (C) HCMEC$_C$ and (D) HCMEC$_D$ immunolabeled for endogenous VWF (blue), P-selectin (P-sel) (red) and tPA (green). Regions in C and D indicated by white boxes are shown in greyscale below colour images. Scale bars: 20μm. Images taken at room temperature using Leica SP2 confocal microscope and software (Mannheim, Germany) equipped with 63x 1.4NA HC PL APO oil immersion objective.

Figure S2

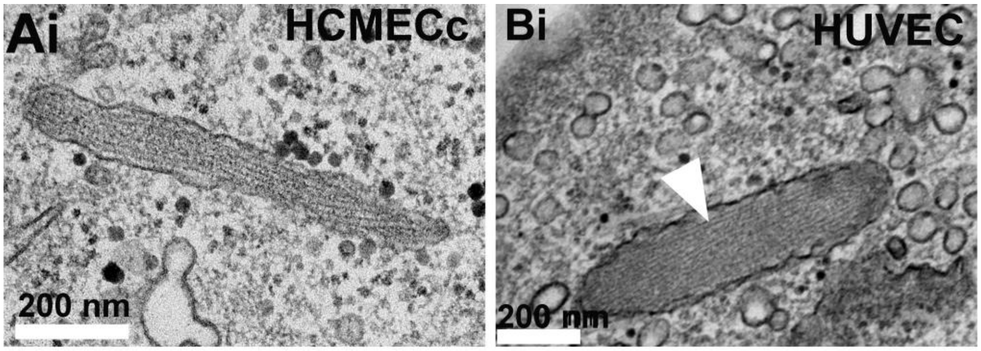


Figure S2 Ultrastructure of mature WPBs in HCMEC$_C$, and HUVEC.
Ai-Bi; en-face thin section (~60nm) TEMs of HCMEC$_C$ (Ai) and HUVEC (Bi) showing mature WPBs with the typical rod-like shape and internal striations (arrow).

Figure S3

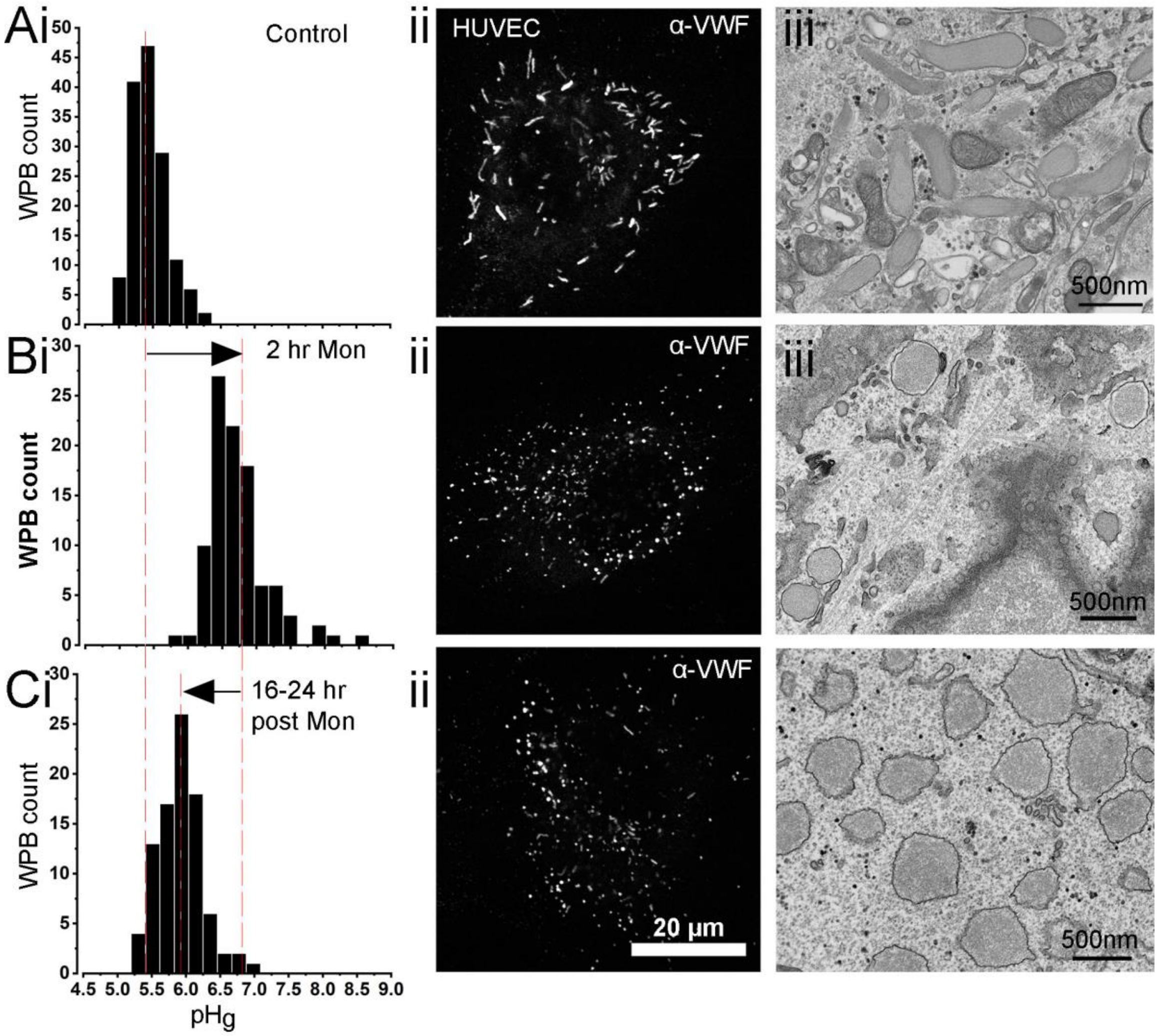


**Figure S3 Intra-WPB pH during disruption of WPB morphology by monensin.**

Ai-Ci; Distributions of the resting intra-organellar pH of WPBs in VWFpp-EGFP expressing HUVEC (black), HUVEC treated with 10µM monensin (Mon) for 2 hours at 37$^{o}$C (Bi, black, n=98 WPBs, mean pH 6.72 ± 0.02), and HUVEC 16-24 hours after removal of Mon (Ci, black, n= 89 WPBs, mean pH 5.90 ± 0.03). Intra-organellar pH was determined from epifluorescence measurements of the steady state fluorescence of EGFP and the maximum fluorescence of the organelle EGFP, obtained after acute application of the weak base ammonium chloride (NH4Cl; 5-10mM), using parameters describing the relationship between EGFP fluorescence and pH determined previously (pKa of 5.84 and nH of 0.74; (Erent et al., 2007)). Aii-Cii show confocal fluorescence images of HUVEC immunolabeled for endogenous VWF under conditions described in Ai-iii. Aiii-Ciii show examples of en-face thin section (~60nm) TEMs of HUVEC under

conditions described in Ai-iii. Vertical red dashed lines show the mean pH in resting HUVEC (left, $HCMEC_D$ (middle) and HUVEC 2 hours post Mon treatment (right). Black arrows show the shift in mean pH of HUVEC WPBs immediately after Mon treatment and following recovery for treatment.

## Citations


Bierings, R., N. Hellen, N. Kiskin, L. Knipe, A.V. Fonseca, B. Patel, A. Meli, M. Rose, M.J. Hannah, and T. Carter. 2012. The interplay between the Rab27A effectors Slp4-a and MyRIP controls hormone-evoked Weibel-Palade body exocytosis. *Blood*. 120:2757-2767.

Erent, M., A. Meli, N. Moisoi, V. Babich, M.J. Hannah, P. Skehel, L. Knipe, G. Zupancic, D. Ogden, and T. Carter. 2007. Rate, extent and concentration dependence of histamine-evoked Weibel-Palade body exocytosis determined from individual fusion events in human endothelial cells. *J Physiol*. 583:195-212.

Hewlett, L., G. Zupančič, G. Mashanov, L. Knipe, D. Ogden, M.J. Hannah, and T. Carter. 2011. Temperature-Dependence of Weibel-Palade Body Exocytosis and Cell Surface Dispersal of von Willebrand Factor and Its Propolypeptide. *PLOS ONE*. 6:e27314.

Knipe, L., A. Meli, L. Hewlett, R. Bierings, J. Dempster, P. Skehel, M.J. Hannah, and T. Carter. 2010. A revised model for the secretion of tPA and cytokines from cultured endothelial cells. *Blood*. 116:2183-2191.
